# Predictive values of early head computed tomography for survival outcome after cardiac arrest in childhood: a pilot study

**DOI:** 10.1038/s41598-021-91628-y

**Published:** 2021-06-08

**Authors:** Kenichi Tetsuhara, Noriyuki Kaku, Yuka Watanabe, Masaya Kumamoto, Yuko Ichimiya, Soichi Mizuguchi, Kanako Higashi, Wakato Matsuoka, Yoshitomo Motomura, Masafumi Sanefuji, Akio Hiwatashi, Yasunari Sakai, Shouichi Ohga

**Affiliations:** 1 Department of Pediatrics, Graduate School of Medical Sciences, Kyushu University, Fukuoka, Japan; 2 Emergency and Critical Care Center, Kyushu University Hospital, Fukuoka, Japan; 3 Department of Medicine and Clinical Science, Graduate School of Medical Sciences, Kyushu University, Fukuoka, Japan; 4 Department of Radiology, Graduate School of Medical Sciences, Kyushu University, Fukuoka, Japan

**Keywords:** Diseases, Medical research, Neurology

## Abstract

Predicting outcomes of children after cardiac arrest (CA) remains challenging. To identify useful prognostic markers for pediatric CA, we retrospectively analyzed the early findings of head computed tomography (CT) of patients. Subjects were non-traumatic, out-of-hospital CA patients < 16 years of age who underwent the first head CT within 24 h in our institute from 2006 to 2018 (n = 70, median age: 4 months, range 0–163). Of the 24 patients with return of spontaneous circulation, 14 survived up to 30 days after CA. The degree of brain damage was quantitatively measured with modified methods of the Alberta Stroke Program Early CT Score (mASPECTS) and simplified gray-matter-attenuation-to-white-matter-attenuation ratio (sGWR). The 14 survivors showed higher mASPECTS values than the 56 non-survivors (*p* = 0.035). All 3 patients with mASPECTS scores ≥ 20 survived, while an sGWR ≥ 1.14 indicated a higher chance of survival than an sGWR < 1.14 (54.5% vs. 13.6%). Follow-up magnetic resonance imaging for survivors validated the correlation of the mASPECTS < 15 with severe brain damage. Thus, low mASPECTS scores were associated with unfavorable neurological outcomes on the Pediatric Cerebral Performance Category scale. A quantitative analysis of early head CT findings might provide clues for predicting survival of pediatric CA.

## Introduction

Intervention for pediatric cardiac arrest (CA) has been a subject of continued study, and the outcomes of pediatric out-of-hospital CA have improved^1,2^. Several prognostic factors of neuroimaging data predict a poor outcome of patients after CA^3^. Computed tomography (CT) is a vital tool in the initial assessment at the emergency room due to its ready availability, short acquisition time and high penetration rate in the medical centers of developed countries. Early head CT is obtained after successful resuscitation to determine the etiology of CA (intracranial hemorrhage, hydrocephalus, tumor and injury) as well as to assess the presence and severity of cerebral ischemia. Hypoxic brain damage shows early CT signs, such as characteristic attenuation values, edema, loss of gray-white matter differentiation and effacement of the cortical sulci^4^.

The gray matter attenuation-to-white matter attenuation ratio (GWR) and Alberta Stroke Program Early CT Score (ASPECTS) are established indices for early ischemic change on CT images and for neurological outcomes after stroke^5–7^. The ASPECTS is determined in 10 regions of the hemisphere of interest for ischemic infarction, including restricted areas supplied by the middle cerebral artery of the affected side. The modified ASPECTS (mASPECTS) covering 12 regions in both hemispheres is used to evaluate the global cerebral damage and prognosis in post-CA adults^7^. The GWR is defined as the ratio of gray to white matter attenuation in Hounsfield units (HU) in 16 regions of interest (ROIs) placed in different brain areas. The simplified GWR (sGWR) in four ROIs is also associated with the outcome of post-CA adults^5^.

These evaluation systems have been mainly applied for adult CA, and prognostic indexes remain to be established for pediatric patients with CA^8^. This may be due to the low incidence of pediatric CA and the etiological heterogeneity (infection, genetic diseases and abuse) in infants and children compared with adults. Recent studies have shown that the GWR was also useful for assessing the neurological outcomes of pediatric CA^9–11^. However, no information is available concerning the utility of ASPECTS in assessing the survival or neurological outcomes after pediatric CA.

In the present study, we investigated whether or not these methods predict the post-30-day survival and neurological outcomes of children after out-of-hospital CA.

## Results

### Demographics of patients

**Figure 1:**
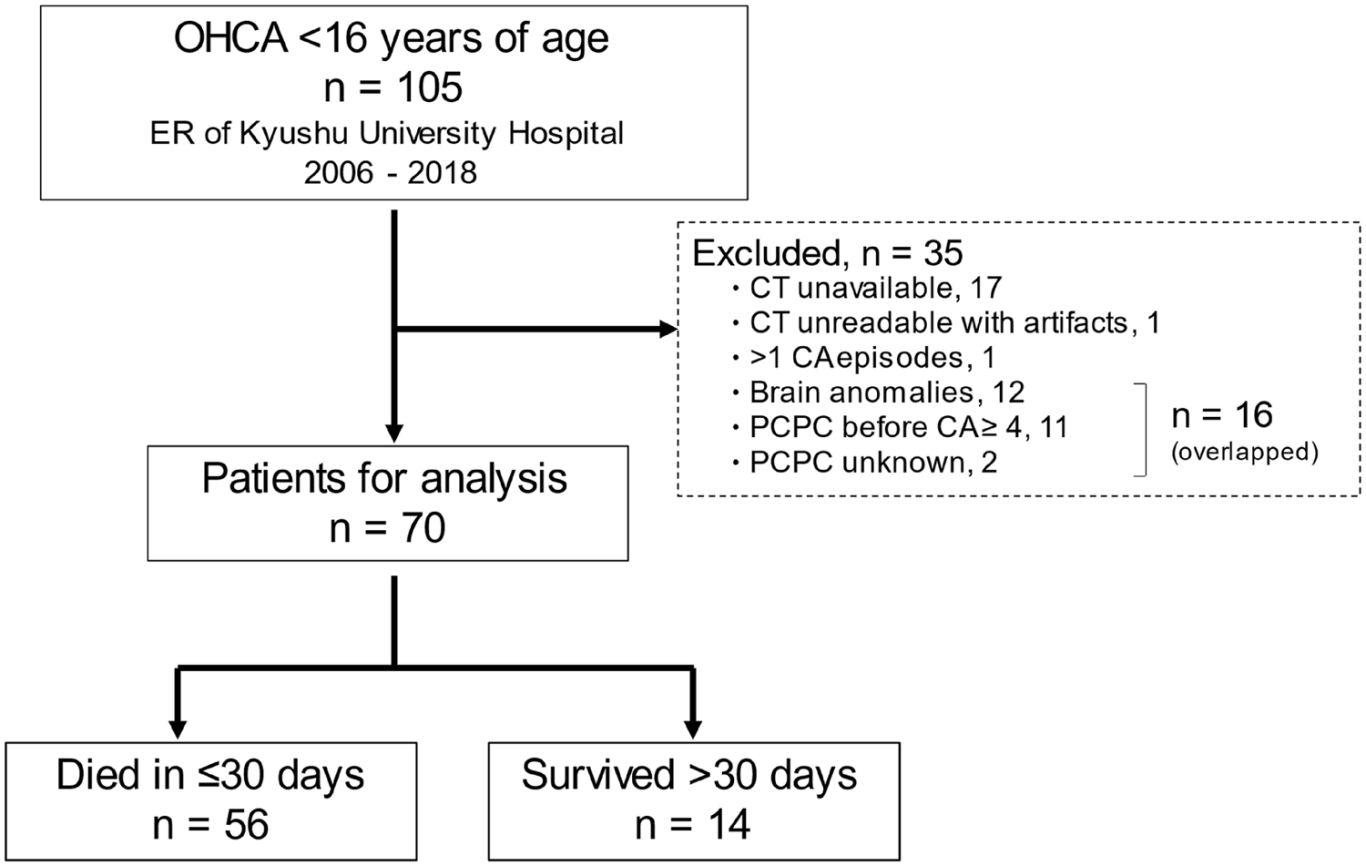
An overview of the present study in a flowchart. Clinical information was collected from a total of 105 pediatric cases with “out-of-hospital cardiac arrest” (OHCA). Ineligible populations (n = 35) were excluded from this study for the indicated reasons: cases without CT data available, only low-quality CT data available, more than one episode of cardiac arrest (CA), brain anomalies, severe neurological disabilities (PCPC ≥ 4) before CA or unknown neurological outcomes. Head CT findings were thus analyzed for the 70 eligible patients. Patients who survived or died were classified according to their outcomes at the primary endpoint of 30 days after CA. PCPC: Pediatric Cerebral Performance Category.

The study population consisted of 70 patients (median age: 4 months ranging 12 days to 163 months, males 46 and females 24) (Tables 1, S1 and Fig. 1). CA was first defined as having either “shockable” or non-shockable rhythms. The non-shockable rhythm included asystole and pulseless electrical activity. Other causes and the number of patients in these categories are summarized in Table S1.

**Table 1:** Demographics of the study population.

	All	Survived > 30 days	Died ≤ 30 days	*p* value
	n = 70	n = 14	n = 56	
Age, months, median, range	4, 0–163	18.5, 0–163	4, 0–118	0.056
Male	46 (65.7)	10 (71.4)	36 (64.3)	0.758
PCPC before CA, median, range	1, 1–3	1, 1–2	1, 1–3	0.841
**Underlying disease**	19 (27.1)	7 (50.0)	12 (21.4)	0.045
CHD	7 (36.8)	1 (14.3)	6 (50.0)	0.044
Non-CHD	12 (63.2)	6 (85.7)	6 (50.0)	
**At detection**				
Bystander CPR	42 (60.0)	9 (64.3)	33 (58.9)	0.770
CPR < 1 h from CA^a^	24 (34.3)	10 (71.4)	14 (25.0)	0.005
Witness of collapse	11 (15.7)	4 (28.6)	7 (12.5)	0.212
Shockable rhythm^b^ (EMS)	3 (4.3)	3 (21.4)	0 (0)	0.007
**On admission**				
ROSC	24 (34.3)	14 (100.0)	10 (17.9)	< 0.001
Shockable rhythm	4 (5.7)	3 (21.4)	1 (1.8)	0.023
**At hospital discharge**				
Median hospital stay (days), range	0, 0–154	63.5, 16–154	0, 0–6	< 0.001
Median PCPC, range	6, 1–6	5, 1–6	6, 6–6	< 0.001

Percentages are shown in parentheses.

EMS, emergency medical service; PCPC, Pediatric Cerebral Performance Category; CA, cardiac arrest; CHD, congenital heart disease; CPR, cardiopulmonary resuscitation; ROSC, return of spontaneous circulation.

^a^Within 1 h between the detection of CA or the last observation of usual conditions and CPR. Data were unavailable for two deceased patients.

^b^Shockable rhythm (ventricular tachycardia or fibrillations) detected by EMS.

Nineteen patients (27.1%) had comorbidities with congenital heart diseases (CHD) or other diseases. Forty-two patients (60%) received bystander resuscitation, and 24 (34.3%) were resuscitated within 1 h after the detection of CA or the last observation of usual conditions. On admission, 24 (34.3%) had obtained return of spontaneous circulation (ROSC), while 4 showed “shockable rhythm” (ventricular tachycardia or fibrillations). The duration of hospital stay ranged from 1 to 154 days. Survival (n = 14) or death (n = 56) outcomes were defined at the primary endpoint of 30 days after CA (Fig. 1). Neurological disabilities among the 14 survivors were assessed according to the Pediatric Cerebral Performance Category (PCPC) scale (Table S2). Nine (64.3%) were defined as being in a coma/vegetative state (PCPC 5), 2 (14.3%) showed severe disability (PCPC 4), 1 (7.1%) had moderate disability (PCPC 2), and 2 (14.3%) had mild disability (PCPC 1).

### Survival factors

The 30-day survivors showed the following features at higher rates than non-survivors: underlying diseases (50.0% vs. 21.4%, *p* = 0.045), CPR performed < 1 h from the last observation (71.4% vs. 25.0%, *p* = 0.005), shockable rhythm at detection (21.4% vs. 0%, *p* = 0.007), shockable rhythm at admission (21.4% vs. 1.8%, *p* = 0.023) and ROSC (100.0% vs. 17.9%, *p* < 0.001) (Table 1).

In contrast, survivors were characterized as showing CHD (14.3% vs. 50.0%, *p* = 0.044, Table S3) and unknown causes of CA (50.0% vs. 83.9%, *p* = 0.012, Table S1) less frequently than non-survivors. The cause of death remained unclear in the majority of non-survivors (81.8–100%) (Table S4). However, multivariable analyses did not identify variables associated with the survival (Table S5).

### Quantitative measurements of brain damage on first-day CT

**Figure 2:**
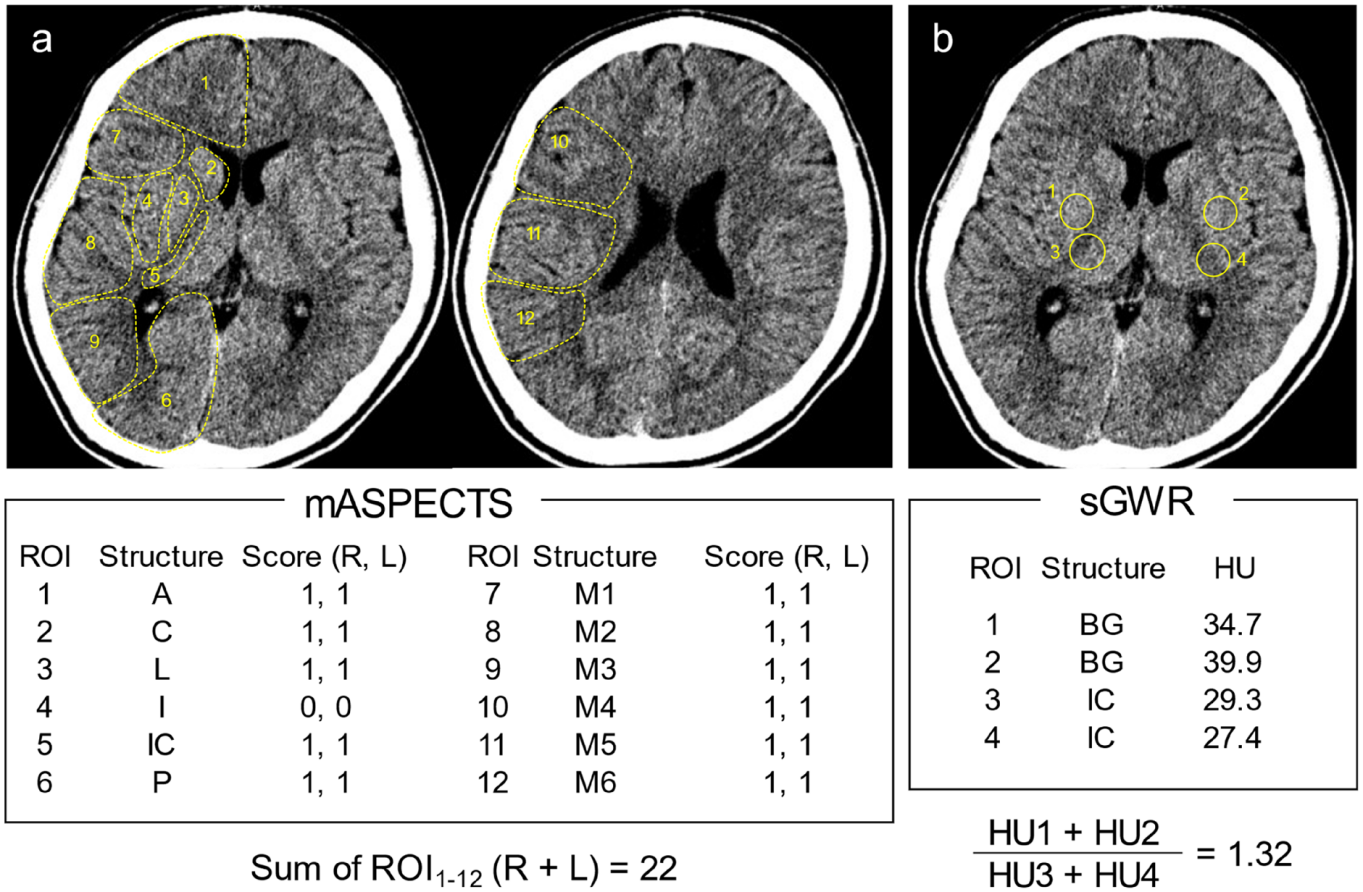
Representative head CT scan in a 13-year-old boy with post-cardiac arrest. (**a**) A schematic presentation of the mASPECTS. Summary of the 24 areas makes the score of 22. (**b**) The formula for calculating the sGWR. Relative HU values of the putamen to those of the posterior limb of the IC equals to 1.32. ROI: region of interest, IC: internal capsule, A: anterior circulation, C: caudate, I: insular ribbon, L: lentiform, M: middle circulation, P: posterior circulation, HU: Hounsfield units, BG: basal ganglia.

To obtain the mASPECTS values, we set 12 ROIs per hemisphere and counted the number of ROIs with (0) and without damaged regions (1) in both hemispheres (Fig. 2a). Thus, the full scale of the mASPECTS ranged from 0 to 24 (mean: 8.7) in the present study. For the sGWR, two and two ROIs were placed in the gray and white matter, respectively. The HU values of the gray matter were standardized with those of the white matter (Fig. 2b, see the Methods for further details). The sGWR values ranged 0.996–1.303 (mean: 1.081) in this study.

**Figure 3:**
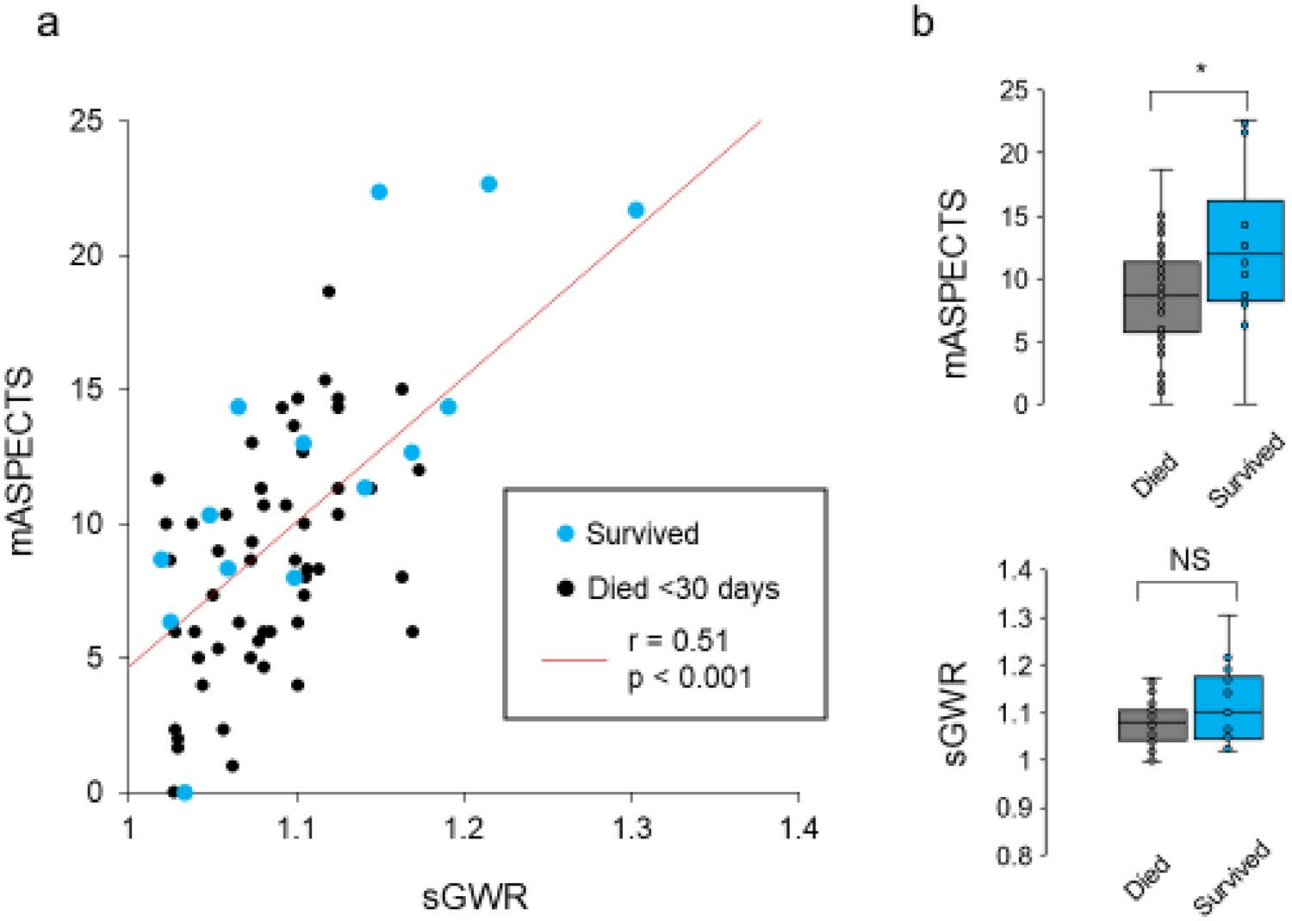
Correlation of the mASPECTS and sGWR scores. (**a**) The scatter plot shows the sGWR (X) and mASPECTS (Y) of survivors (blue dots) and non-survivors (black). Spearman’s correlation coefficients are shown in the plots. (**b**) The box plots represent the mASPECTS (upper) and sGWR (lower) scores of survivors and non-survivors. NS, not significant.

To test whether or not these brain damage-scoring systems provided consistent results, we first compared the mASPECTS and sGWR values from the 70 total patients. We verified that the values in these 2 scales were correlated with each other (r = 0.51, *p* < 0.001, Fig. 3a). We then examined whether or not the 14 survivors and 56 non-survivors showed markedly different values for the mASPECTS or sGWR. We found that survivors had higher mASPECTS values than non-survivors (Mann–Whitney *U*-test, *p* = 0.035; Fig. 3b, upper). However, no such difference was observed in the sGWR (*p* = 0.209) (Fig. 3b, lower).

Although the data above suggest that a higher mASPECTS predicts favorable outcomes in the 30-day survival after CA, the apparent association might merely reflect that 7 of the 14 survivors (50%) were ≥ 12 months of age. To clarify this issue, we explored whether or not the age of the patients affected the mASPECTS and sGWR values and found that the older patients (≥ 12 months of age) had higher mASPECTS and sGWR values than the younger ones (*p* = 0.011 and *p* < 0.001, respectively; Figure S1).

**Figure 4:**
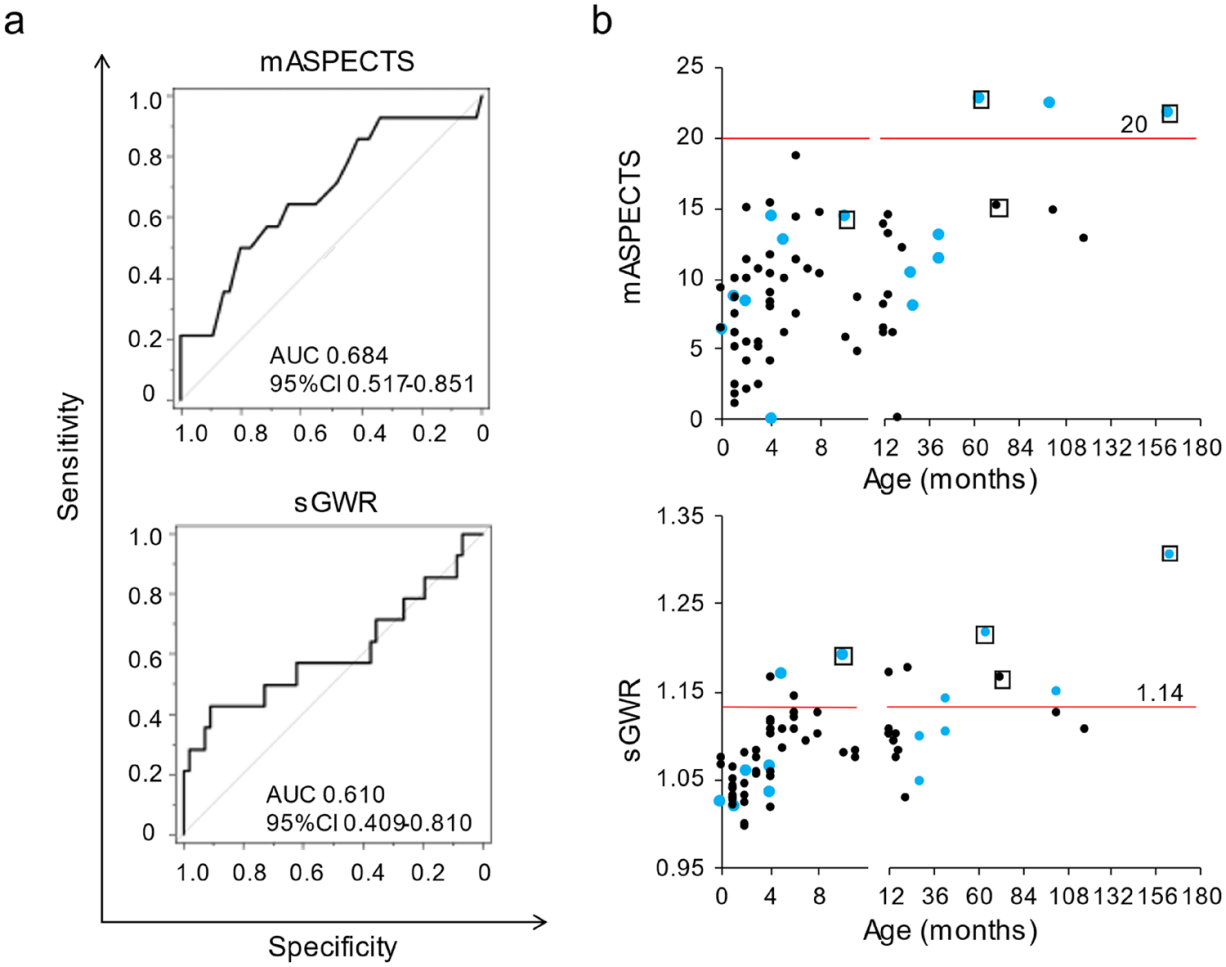
Prognostic values of the mASPECTS and sGWR for 30-day survivors. (**a**) Receiver operating characteristic (ROC) curves for the mASPECTS (upper) and sGWR (lower). No significant differences in the areas under the ROC curve between the 2 systems are observed (*p* = 0.32). (**b**) The mASPECTS (upper) and sGWR (lower) scores against the age of patients at CA. More survivors (blue) than non-survivors (black) are plotted above the cut-off lines (mASPECTS ≥ 20 and sGWR ≥ 1.14). Fisher’s exact tests (mASPECTS; *p* = 0.007, sGWR; *p* = 0.006). Open squares denote the four patients with shockable rhythm (ventricular tachycardia and/or fibrillation).

To identify 30-day survivors, ROC curves were drawn for survival or non-survival outcomes with the mASPECTS, resulting in an area under the curve (AUC) of 0.684 (95% CI 0.517–0.851). In contrast, the sGWR showed an AUC of 0.610 (95% CI 0.409–0.810) (Fig. 4a). The AUCs did not differ significantly between the two age groups (≥ 12 and < 12 months old) for either the mASPECTS or sGWR (Figure S2). However, the older group showed higher AUCs than the younger group in both systems (0.703 vs. 0.585 in the mASPECTS; and 0.659 vs. 0.522 in sGWR).

According to the ROC data above, we set the cut-off value for the mASPECTS to 20. Three patients were classified as having an mASPECTS ≥ 20, and all three survived (Fig. 4b). Thus, the survival rate of the over-the-cut-off patients was higher than that of the under-the-cut-off patients (100% vs. 19.6%, *p* = 0.007, Fisher's exact probability test). The sensitivity and specificity of the mASPECTS for the 30-day survival were 21.4% and 100%, respectively. Similarly, we defined the cut-off value at 1.14 on the sGWR system. Six out of 11 patients (54.5%) with an sGWR ≥ 1.14 survived to 30 days after CA, whereas only 8 of 59 (13.6%) with an sGWR < 1.14 were 30-day survivors (*p* = 0.006, Fig. 4b). A higher sensitivity (91.1%) but lower specificity (42.9%) than the mASPECTS was observed with the sGWR system for identifying 30-day survivors.

When we investigated the demographic features of patients who fit these cut-off indexes more closely, all three patients with an mASPECTS ≥ 20 were children ≥ 12 months old (Figs. 4b, S3). Consistently, 7 of the 11 patients (64%) with an sGWR ≥ 1.14 belonged to this age group (≥ 12 months). Furthermore, 2 of the 4 patients with shockable rhythms (a favorable factor for 30-day survival, Table 1) showed an mASPECTS ≥ 20, and both survived for 30 days after CA. In contrast, all 4 patients with a shockable rhythm had an sGWR ≥ 1.14 (Fig. 4b). However, one of them died within 30 days after CA. Thus, the mASPECTS more stringently predicted survivors after CA than did the sGWR (Figure S3).

### Clinical profiles of non-survivors

Ten patients with ROSC died on days 1–6 after admission (median 1 day, Table 1). Four presented with multiple organ dysfunction, which is a typical symptom of post-resuscitation syndrome (PRS)^8^; one showed cardiac dysfunction due to arrhythmia; and the remaining five died of underlying illness or unidentified causes (Figure S4a). These 10 patients did not show significant difference in the mASPECTS scores compared with non-survivors without ROSC (Figure S4b). The mASPECTS scores did not differ between non-survivors who died within 24 h (n = 5) and those who survived ≥ 24 h after CA (n = 5; Figure S4c), either.

Patients with unknown causes of CA (n = 54) were included in this study. Of these 54 patients, 43 (79.6%) died within 24 h, and 4 (7.4%) survived the first day and died by Day 6 (Figure S5a). Seven patients (13.0%) with unknown causes of CA survived to 30 days after CA (Table S1). The survival duration did not differ markedly between non-survivors with known (n = 9) and unknown causes of CA (n = 47; Figure S5a).

Causes of death for the 47 non-survivors included PRS (n = 4; Figure S4a). Thus, both cardiac and non-cardiac mechanisms were considered for the underlying causes of CA in this category. However, even with a close inspection of the data based on an evaluation of their medical charts and autopsy findings, we were unable to determine the direct causes of CA. We therefore subclassified their CA-prone backgrounds according to their history: congenital heart disease (CHD, n = 5), non-CHD (n = 6) and no diseases (n = 36). We observed a longer survival for patients with non-CHD than for the other two subgroups of non-survivors (Figure S5b). Thus, “unknown causes of CA” were considered to include various conditions from reversible to irreversible stages after the onset of CA. In line with this concept, we verified that known or unknown causes of CA did not affect the mASPECTS scores (Figure S5c). Similarly, no marked difference in the mASPECTS scores was noted among the 47 non-survivors without known causes of CA when they were sub-grouped according to the history of CHD, non-CHD and no underlying diseases (Figure S5d).

### Relationship of the mASPECTS with magnetic resonance imaging (MRI) findings

Because the mASPECTS segregated 30-day survivors from non-survivors with higher specificity than did the sGWR (≥ 1.14), we further explored whether or not the mASPECTS might also provide predictive values for neurological disabilities of survivors.

**Figure 5:**
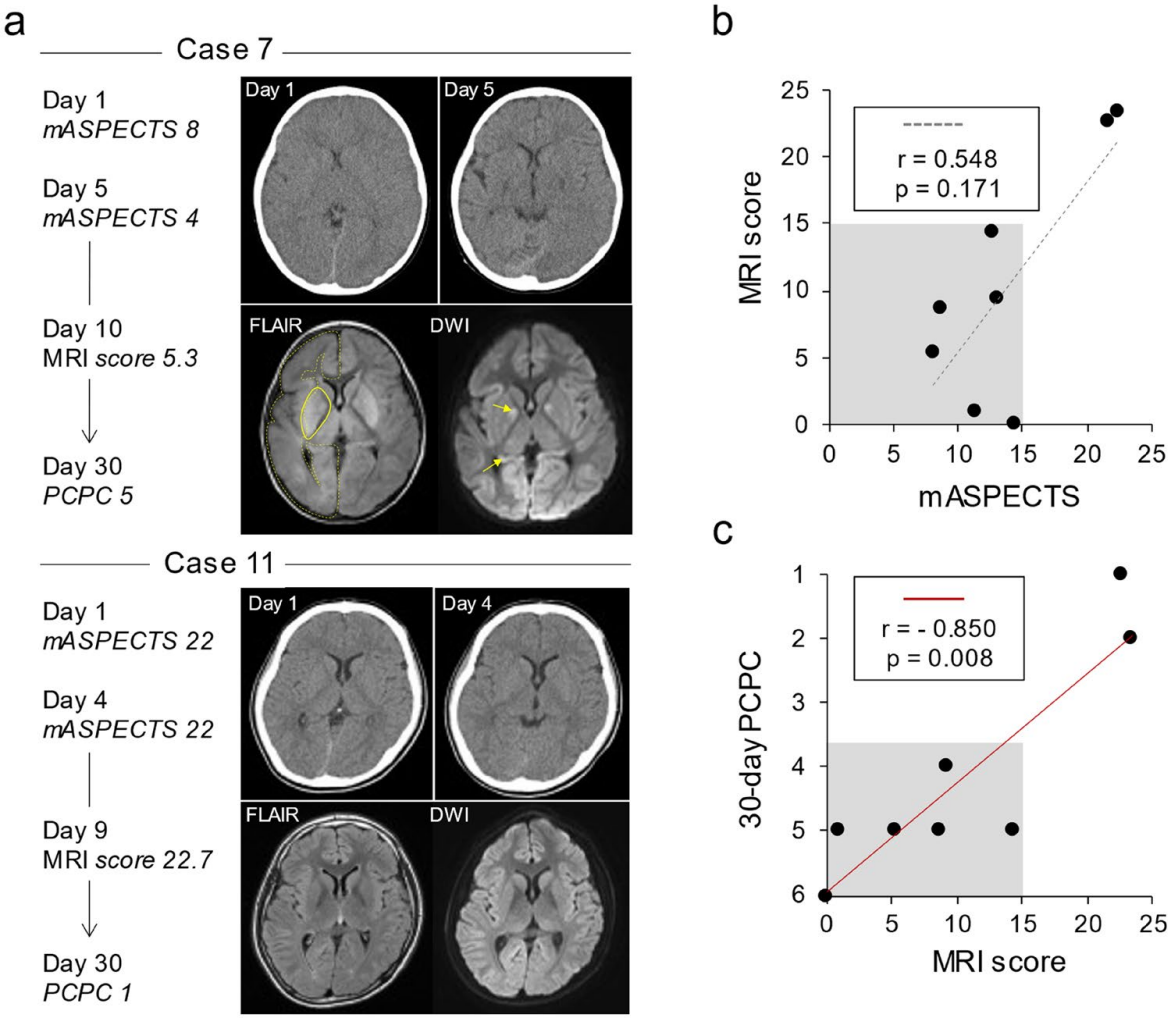
Follow-up MRI findings and neurological disabilities of the 30-day survivors. (**a**) Time courses of two representative cases with different mASPECTS and neurological outcomes. Note that Case 7 (upper) showed more severe brain damage and neurological disability at day 30 than Case 11 (lower). In the right panels, the first CT (left upper), second CT (right upper) and follow-up MRI (FLAIR and diffusion-weighted image, DWI) findings are shown. Yellow lines and arrows indicate the regions with abnormal signals on FLAIR imaging and DWI, respectively. Only the right hemispheres are marked for clarity. (**b**) Relationship between the mASPECTS and MRI scores among the 8 survivors who received MRI within 30 days after CA. Severe brain damage (mASPECTS < 15, shaded) corresponded to a low MRI score (< 15). (**c**) The 30-day PCPC scores of 8 cases. Shaded areas indicate severe neurological disabilities (PCPC ≥ 4) for patients with MRI scores < 15. The MRI score and 30-day PCPC were significantly correlated (r = − 0.850, *p* = 0.008).

To this end, we next determined the brain damage scores using MRI. Follow-up MRI was performed for 11 of 14 survivors. Of those, 8 had MRI data prior to the endpoint of 30 days after CA (Figure S6). For a comparative analysis of brain damage on MRI, 24 ROIs were placed in areas identical to those of the mASPECTS. For example, 2 representative cases (#7 and #11) marked the mASPECTS scores, 8 and 22, respectively (Fig. 5a). Repeated CT consistently showed that the brain of Case 7 was more severely injured than that of Case 11. The quantitative measurements of follow-up MRI confirmed the presence of profound brain damage in Case 7 compared to Case 11. In agreement with the imaging findings, Case 7 showed a higher degree of neurological disability (PCPC 5) than did Case 11 (PCPC 1).

Because MRI detected parenchymal lesions more sensitively than CT, no significant correlation was observed between the mASPECTS and MRI scores of the eight cases tested herein (Figs. 5b, S7). However, we found that an mASPECTS < 15 corresponded to severe lesions on MRI (MRI score < 15). Thus, our results indicated that the mASPECTS on the first CT scan successfully indicated severe brain damage on MRI. Notably, the MRI scores of these 8 patients were correlated with their 30-day PCPC scores (*p* = 0.008, Spearman’s rank correlation coefficient, r = − 0.850; Fig. 5c). Thus, the MRI scores were reliable indexes for neurological disabilities among the eight survivors.

### The first CT scores as predictive values for post-CA neurological disabilities

**Figure 6:**
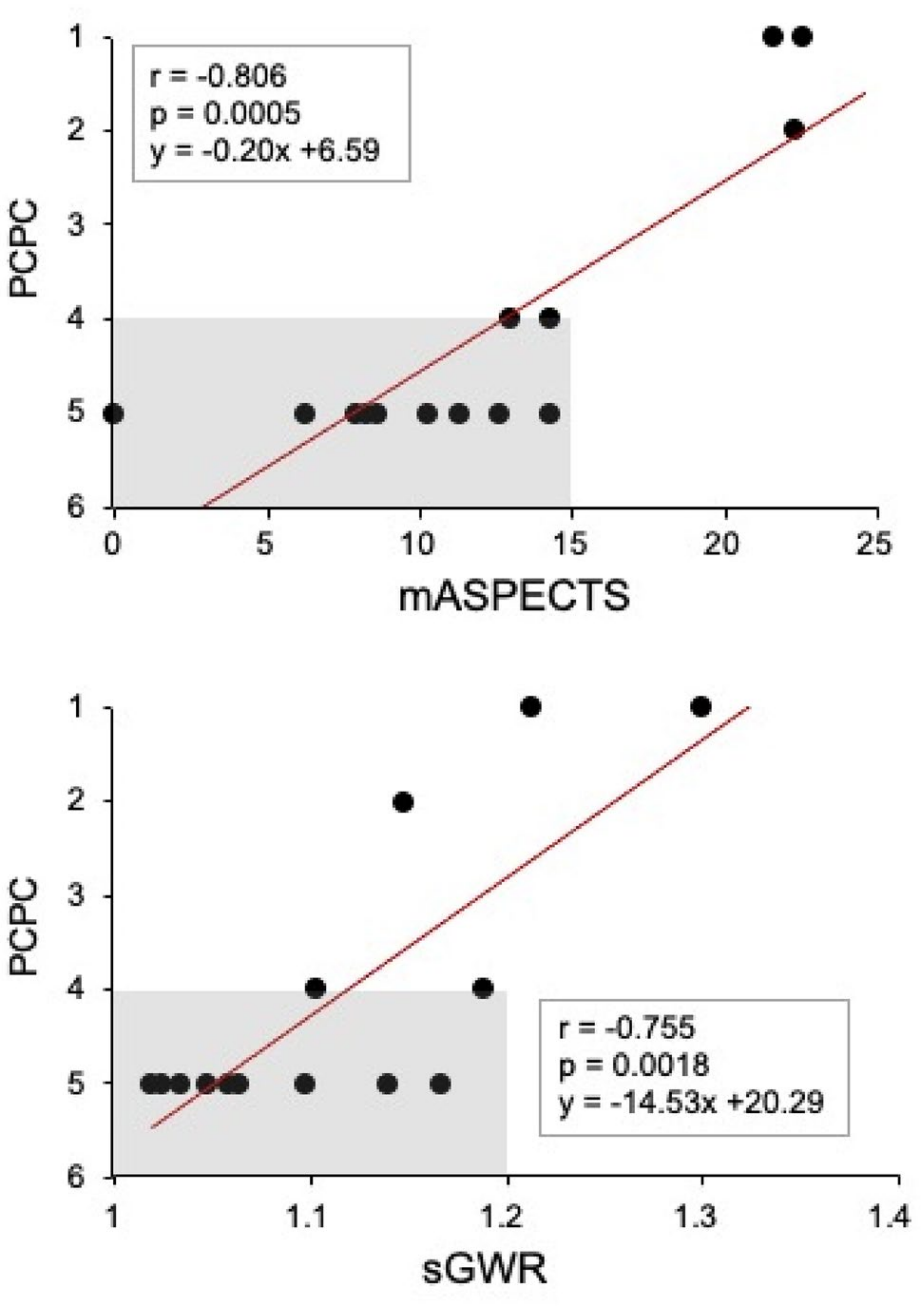
Correlation of the mASPECTS and sGWR with PCPC at 30 days after CA. Severe neurological disabilities (higher PCPC scores) among 14 survivors are correlated with lower mASPECTS (r = − 0.81, *p* = 0.0005) and sGWR values (correlation coefficient r = − 0.76, *p* = 0.0018). Shaded areas highlight the 11 patients with severe neurological disabilities (PCPC ≥ 4).

Finally, we investigated whether or not the first CT scores were useful for predicting the neurological outcomes of 14 survivors. To this end, we analyzed their correlations with 30-day PCPC and found that the PCPC scores were inversely correlated with the brain damage indexes determined with the mASPECTS (*p* = 0.0005) and sGWR (*p* = 0.0018) on the first CT (Fig. 6). A multiple linear regression analysis showed that the PCPC scores of the survivors were correlated with the mASPECTS (β = − 0.14, *p* = 0.028) but not with the sGWR (β = − 5.71, *p* = 0.224). Neither a covariance analysis nor correlation coefficients showed a marked difference in the slope index between the two scoring systems.

Among the 7 patients with CHD, one survived to 30 days after CA, two died on Day 1 and Day 5, and the remaining four died within 24 h. Notably, the survivor (Case 10 in Figure S6) with CHD had an mASPECTS of 22.3, which was higher than the values (0–15) of non-survivors (CHD, Figure S5d). Consistent with the mASPECT score, the only survivor with CHD showed mild neurological impairment. These data support the notion that the mASPECTS might be a useful system for predicting the survival and neurological outcomes after CA in patients with CHD.

## Discussion

In the present study, we showed that the first-day CT findings provided useful parameters for the quantitative analysis of the brain damage sustained after CA in pediatric patients. We first demonstrated that the mASPECTS functioned as a reliable score of brain damage after CA as did the sGWR, a more prevalent parameter in pediatric critical care medicine^9–11^. The mASPECTS system for children may compensate for the disadvantages observed with the sGWR in stringently predicting the survival of and neurological disabilities among survivors after CA. As suspected, however, either mASPECTS or sGWR did not necessarily discriminate survivors from non-survivors at a sufficient sensitivity and specificity for infants (< 12 months of age).

Analytical difficulty was also related to the wide variety of etiological backgrounds of CA in our study. Indeed, previous studies have reported the rate of unknown cause of CA to be 25–67%^12,13^, although our present study included a higher rate of patients with unknown cause of CA (54/70; 77%). This may be due in part to lower rates of sudden infant death syndrome than the previous studies (4.0% vs. 13–63%) and the fact that we performed an autopsy for only 11 patients (19.6% of non-survivors).

Several variables are known to be associated with the outcomes of pediatric CA, including shockable rhythm^8^. In our study population, neither increased rates of witnessed collapse nor bystander CPR were indicated as significant survival factors. Furthermore, a multivariable analysis identified no useful factors for predicting the post-30-day survival among the variables deemed significant on a univariate analysis. The relatively long interval from the last observation to CPR and the sample size may account for the discrepancy in the two variables from the results of previous studies^8^. In contrast, we found that 3 of 4 patients with shockable rhythm and an mASPECTS > 20 or sGWR > 1.14 survived. This may suggest that shockable rhythm is a more useful factor associated with the survival if combined with early head CT scores.

Both the mASPECTS and sGWR are used to assess the neurological outcomes of out-of-hospital CA in adults. The ASPECTS has served as a useful marker for the neurological outcomes, having an AUC of 0.88–0.92 in adults^4,7,14^. When the cut-off of the mASPECTS was set at 13, the sensitivity and specificity for poor neurological outcomes were reported to be 66% and 100%, respectively^7^. For pediatric patients, the ASPECTS has been used to predict the outcomes after stroke with MRI^15–18^. Thus far, however, no previous studies have targeted children with out-of-hospital CA or analyzed their data based on head CT findings. We found that the AUC for the post-30-day survival was 0.68 for the mASPECTS in children, which was lower than that in adults. In contrast, the correlation coefficients of the mASPECTS (r = − 0.81) and sGWR (r = − 0.76) for the neurological outcome were deemed to be satisfactory. Multiple linear regression analyses indicated a distinctive pattern concerning the association of PCPC with mASPECTS from sGWR. Taken together, the mASPECTS is considered to be a useful marker for the outcomes of pediatric CA.

More reports have indicated the utility of the GWR as a prognostic marker of out-of-hospital CA adults^5,19–26^ than have indicated its negative utility^4,27^. A systematic review indicated that the GWR-based method obtained an AUC of 0.93 for prognosing neurological outcomes^6^. The cut-off values of the GWR for predicting poor neurological outcomes with 100% specificity ranged from 1.06 to 1.21^6,19–26^. However, only a few reports have shown the utility of GWR for pediatric CA, and those studies showed lower AUCs (0.74–0.82) than did adult studies^9–11^. We obtained an AUC of 0.61 for the sGWR in the present study, which was even lower than those values in previous reports. In subgroup analyses, the AUC for infants (< 12 months of age) further decreased to 0.522, while that for children (1–15 years of age) increased to 0.659. Thus, the lower AUC in our study may be due in part to the skewed age distribution of participants toward a younger age. In contrast, the cut-off value of 1.14 for the sGWR was consistent with the values determined in previous studies (1.08–1.28)^9–11^. The GWR physiologically increases with age in children^10^ due to differences in the growth between gray and white matter, as gray matter develops during the first two years of life, followed by the development of the white matter^28,29^.

As a predictive marker of survivors after CA, the mASPECTS had a higher specificity (100%) than did the sGWR (42.9%) but a lower sensitivity (21.4%; sGWR = 91.1%). These data reflect the fact that the mASPECTS score may vary among researchers, while only a stringent level of agreement yields the excellent score (> 20). In contrast, the sGWR provides consistent results in the quantitative assessment of brain damage, but the cut-off value with the fixed ROIs serves as but one of many predictive markers. Because the present study recruited only three survivors who had neurologically mild impairment (PCPC 1 or 2), the mASPECTS needs to be evaluated further in more survivors in this category. A prospective analysis of the mASPECTS and MRI findings might provide clues for identifying more accurate markers predicting the neurological outcomes of patients after CA.

Several limitations associated with the present study warrant mention. First, the study was performed with a retrospective design and a single-institution setting. Recapitulating our data in independent facilities might further strengthen the utility of both the mASPECTS and sGWR in broad categories of children with out-of-hospital CA. Second, an interrater difference was noted. As illustrated in earlier studies, the regions most vulnerable to hypoxic injury in the brain depend on the age: the deep gray matter in neonates, the diffuse gray matter and regions supplied by the posterior cerebral arteries in post-neonatal infants and the deep gray matter and cerebellum in older children^30^. These aspects make it difficult to interpret pediatric CT images manually. An artificial intelligence-based approach might be an alternative method of standardizing the structural variability and differences in myelination^31–33^. Third, the underlying etiologies, specific genetic backgrounds and main trigger of the onset of pediatric CA remained unclear in the present study. Combined analyses of other predicting variables with the mASPECTS and sGWR might shed some light on these issues. Finally, MRI was performed at variable points for 11 survivors after CA in this study. As acute-phase diffusion-weighted imaging is an established modality for prognosing neurological outcomes^34^, the early findings remain to be characterized in future studies of survivors after CA. Alternatively, time-adjusted MRI analyses may provide an index score of mASPECTS that is associated with favorable neurological outcomes among survivors.

These data may not support the notion that early-phase CT predicts the survival or death after CA. We also understand that head CT does not necessarily indicate systemic inflammation, metabolic deregulation or other conditions. Thus, the value of head CT might be further clarified by determining why a patient died despite having good sGWR and mASPECTS values and why others showed severe neurological disabilities despite promising mASPECT scores supporting otherwise favorable outcomes. Such efforts may help clarify the unique role of head CT in an optimized system for monitoring patients with ROSC.

In conclusion, early head CT may be useful for predicting survival after CA in childhood. Future research is needed to determine if findings on early head CT are associated with longer term neurological outcomes in surviving children.

## Methods

### Ethics

This study was started after obtaining approval from the Kyushu University Hospital Institutional Review Board (#2019–397). All procedures in the acquisition of clinical data, analyses and preparation of this manuscript were conducted in strict compliance with the institutional guideline. Informed consent was waived due to the retrospective nature of this study. The opt-out method on our official website was used, which was approved by the Kyushu University Hospital Institutional Review Board.

### Study design and populations

A total of 105 non-traumatic out-of-hospital CA patients were enrolled in the present study. These participants were < 16 years of age and visited the emergency room of Kyushu University Hospital (KUH) between August 1, 2006, and July 31, 2018. This tertiary hospital has an extracorporeal membrane oxygenation (ECMO) center, pediatric intensive-care unit (ICU) and high-level pediatric medical center and is located in the northern part of Kyushu Island, which covers an area of 42,231.5 km^2^ and had an estimated population of 12,795,884 on January 1, 2020.

The inclusion criteria were patients who had a performance score of ≤ 3 on the Pediatric Cerebral Performance Category (PCPC) scale (Table S2)^35^, who had received head CT within 24 h after CA and who were not transferred from other hospitals. Thirty-five patients were excluded from this study because they had more than one CA episode, an abnormal structure of the brain, or unreadable CT scans due to artifacts (Fig. 1). Thus, the remaining 70 patients were subjected to the quantitative analysis of CT scans on admission, the survival, and the neurological outcomes.

The details of underlying disease are summarized in Table S6. Withdrawal and withholding of treatments were not discussed or carried out for non-survivors in this study.

### Variables

Clinical data were prospectively collected from the medical charts according to the Utstein guidelines^36^. Other data, including underlying disease, 30-day survival status and 30-day PCPC, were retrospectively collected from medical charts. Shockable rhythm includes ventricular fibrillation and pulseless ventricular tachycardia. Non-shockable rhythm includes asystole and pulseless electrical activity (Table S1). When the cause of CA was not clarified based on the history, physical examination findings, blood tests, or imaging, the cause of CA was defined as “unknown”. An autopsy was performed for 11 patients, including 3 with CA who had suspected—but no robust evidence for—respiratory causes or cardiogenic events. These patients were included in the category of “unknown” because they were older than 12 months of age. Initial early brain CT was performed within 24 h after CA. The duration of the hospital stay was recorded as of March 2020.

### Measurement of the mASPECTS and sGWR

Three researchers (YW: pediatrician, MK: neurologist, and KT: pediatric emergency physician) reviewed the initial unenhanced brain CT images. The patient information other than age was removed. The researchers independently measured the mASPECTS and sGWR according to the established methods and made agreements with the results of mean values^5,7^. The physicians who treated the patients were excluded from the quantitative measurement.

The mASPECTS was calculated as previously described (Fig. 2a)^7^. In brief, 12 ROIs were set in each hemisphere: 3 regions at the subcortical area/basal ganglia in the caudate head, lenticular nucleus and posterior limb of the internal capsule; and 9 cortical regions according to the territory of the middle, anterior and posterior cerebral artery. In total, 24 regions across both hemispheres were analyzed. When early ischemic findings, such as parenchymal hypoattenuation, cortical swelling, and loss of gray and white matter differentiation, were present in an ROI, the score was 0 points. In contrast, when there were no early ischemic findings, the score was 1 point. The mASPECTS was calculated as the sum of the points of all 24 ROIs (maximum: 24).

The sGWR was obtained based on the relative HU value of the gray matter to that of the white matter: sGWR = bilateral putamen / posterior limb of the internal capsule at the basal ganglia level (Fig. 2b). The HU values were obtained by dividing the total sum of the ROI of 0.1–0.15 cm^2^ by the area.

### The quantitative analysis of MRI findings

Twenty-four ROIs were placed in areas identical to those of the mASPECTS (Fig. 2a). Conventional MRI of T1-weighted, T2-weighted, fluid attenuated inversion recovery (FLAIR) and diffusion-weighted imaging (DWI) was used for the evaluation. The ROI was defined as 0 (damage +) when any lesions were detected or 1 (damage -) when no lesions were identified. Thus, an intact brain scored 24 points in this MRI scoring system.

Two board-certified pediatric neurologists (MS and YS) and one neuroradiologist (AH) independently reviewed MRI data and came to an agreement concerning the mean score of each patient (Figure S7).

### Statistical analyses

Differences between the two groups were analyzed using the Mann–Whitney *U*-test, Fisher's exact probability test according to continuous and categorical variables. A multiple logistic regression analysis analyzed the factors potentially associated with the survival of the patients. Spearman's rank-sum test was used for the association study. A multiple linear regression analysis assessed the association of the post-30-day PCPC with the mASPECTS or sGWR. A covariance analysis compared the slope index of the regression equations. An intraclass correlation coefficient was used as a measure of concordance. To set the cut-off values for scores to discern survivors from non-survivors and to determine the sensitivity and specificity, a receiver operating characteristic (ROC) analysis was conducted. The cut-off value was calculated so that the sum of the sensitivity and specificity was the highest on the ROC curve. A *p*-value < 0.05 was considered to be statistically significant. EZR version 1.36 (Saitama Medical Center, Jichi Medical Univ., Saitama, Japan, The R Foundation, Vienna, Austria) was used for the data analyses^37^.

## Supplementary Information

Supplementary Information.

## Data Availability

All data generated or analyzed during this study are presented in this article and Supplementary Information files.
