# Supplementary material for: Predictive values of early head computed tomography for survival outcome after cardiac arrest in childhood: a pilot study
Source: Sci Rep. 2021 Jun 8;11:12090. doi: 10.1038/s41598-021-91628-y (PMC8187472; doi:10.1038/s41598-021-91628-y)
Supplement: Supplementary file 1 — Supplementary Information. [file 41598_2021_91628_MOESM1_ESM.pdf]

Supplementary material

Predictive values of early head computed tomography for survival outcome after cardiac arrest in childhood: a pilot study

Kenichi Tetsuhara, Noriyuki Kaku, Yuka Watanabe, Masaya Kumamoto, Yuko Ichimiya, Soichi Mizuguchi, Kanako Higashi, Wakato Matsuoka, Yoshitomo Motomura, Masafumi Sanefuji, Akio Hiwatashi, Yasunari Sakai, Shouichi Ohga

Corresponding author: Noriyuki Kaku kakunori@pediatr.med.kyushu-u.ac.jp

This file contains the following materials:

- Supplementary figures and legends (Figure S1 – S7)
- Supplementary tables (Table S1 – S6)

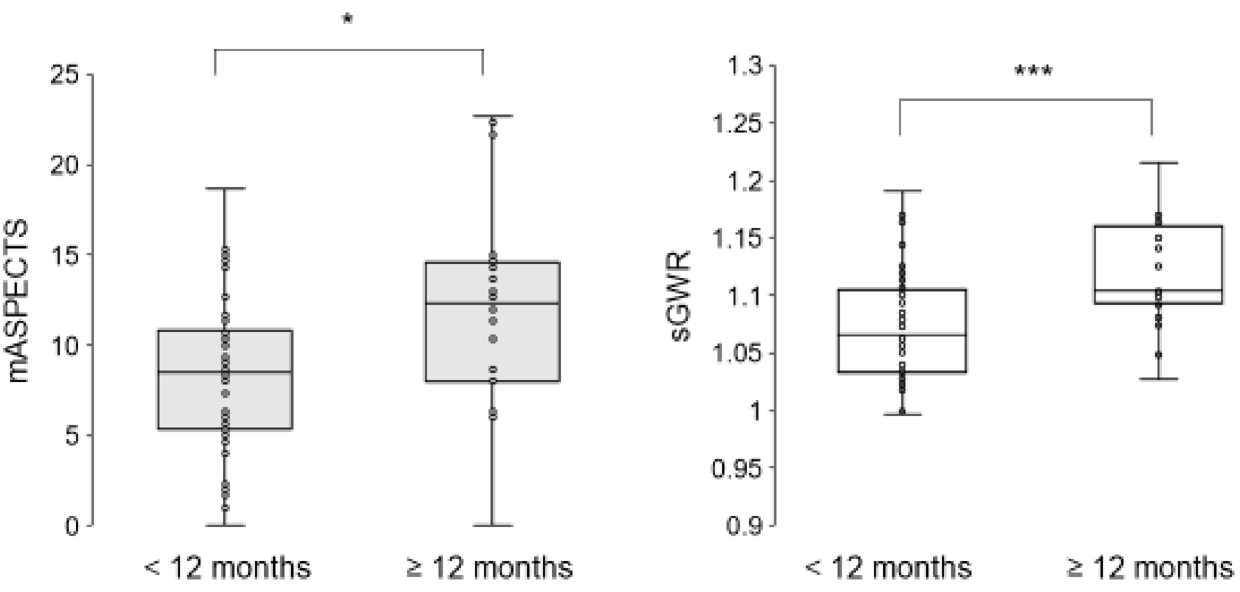

Figure S1. Box-dot plots for mASPECTS and sGWR scores of patients in two age groups. Note that older patients (≥12 months, n = 20) marked higher scores both in mASPECTS and sGWR than younger patients (<12 months, n = 50). 8.5 *vs.* 12.3 in mASPECTS (p = 0.011); and 1.066 *vs.* 1.104 in sGWR (p = 0.00098). *p <0.05, ***p <0.001 (Mann-Whitney U-test).

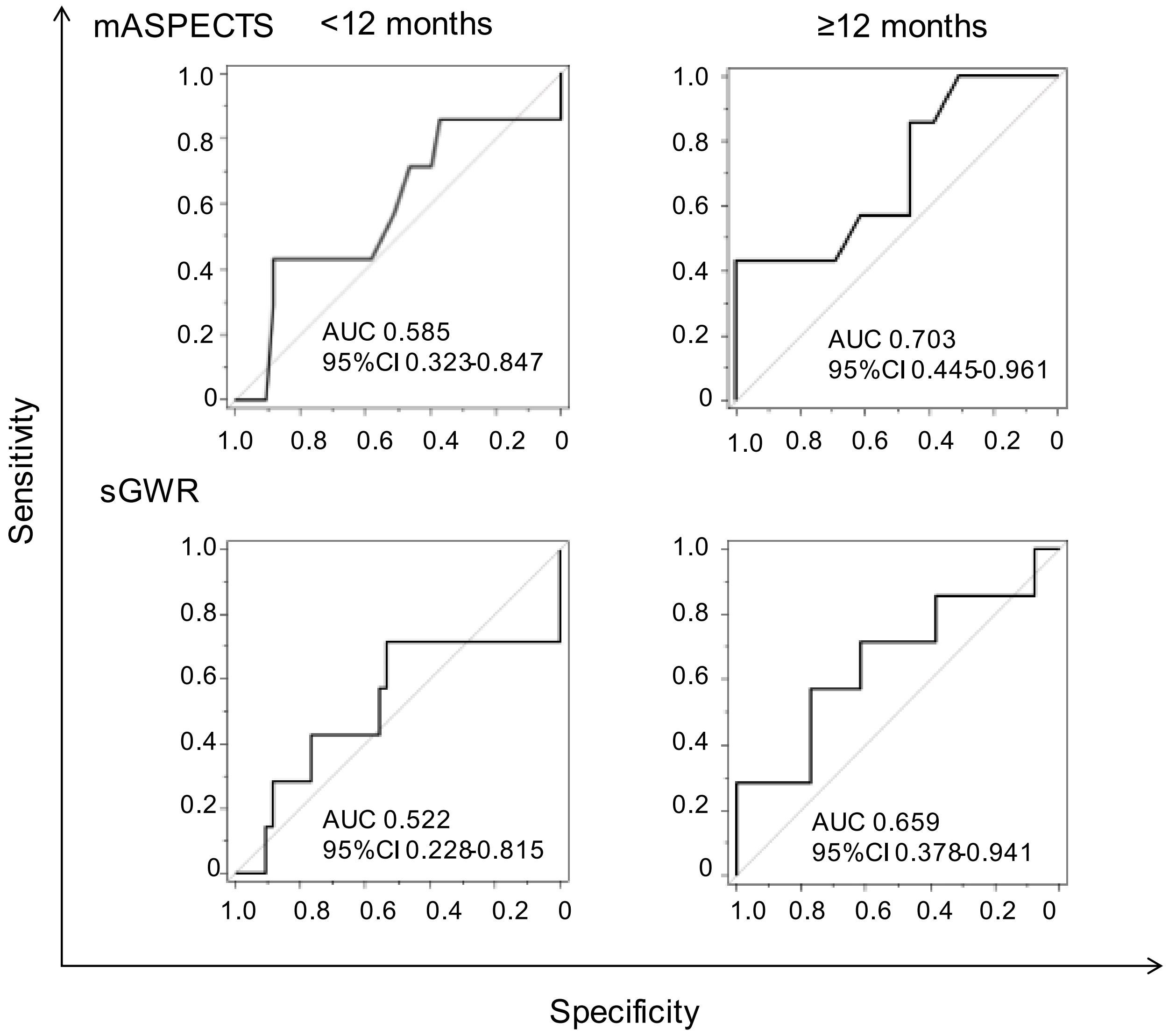

Figure S2. ROC curves for sGWR and mASPECTS in the two age groups.

No significant differences were observed in AUC values between mASPECTS (upper) and sGWR (lower) for the two age groups: Age <12 months ($p = 0.80$, left) and age ≥12 months ($p = 0.73$, right).

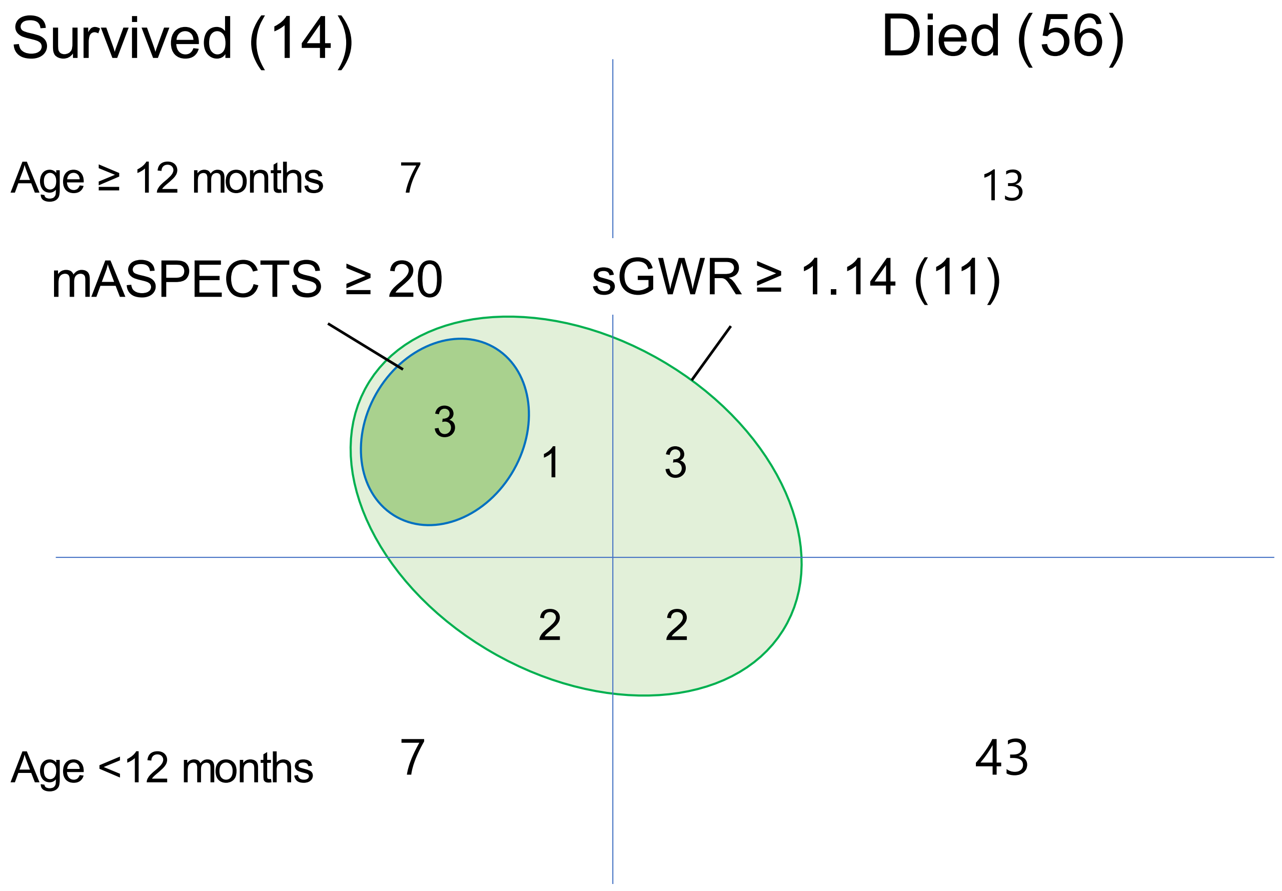

Figure S3. An overview of patients on diagnostic categories.

Seventy patients are classified into "Survived" (n = 14) and "Died" (n = 56) groups. According to the age of CA, they were subclassified into the age groups of ≥12 months (n = 7 and 13) and <12 months (n = 7 and 43), respectively. The Venn diagrams overlaid on the four quadrants show subgroups of patients who passed mASPECTS ≥20 (n = 3) and sGWR ≥1.14 (n = 11) criteria. Note that mASPECTS more stringently predicted survivors than sGWR.

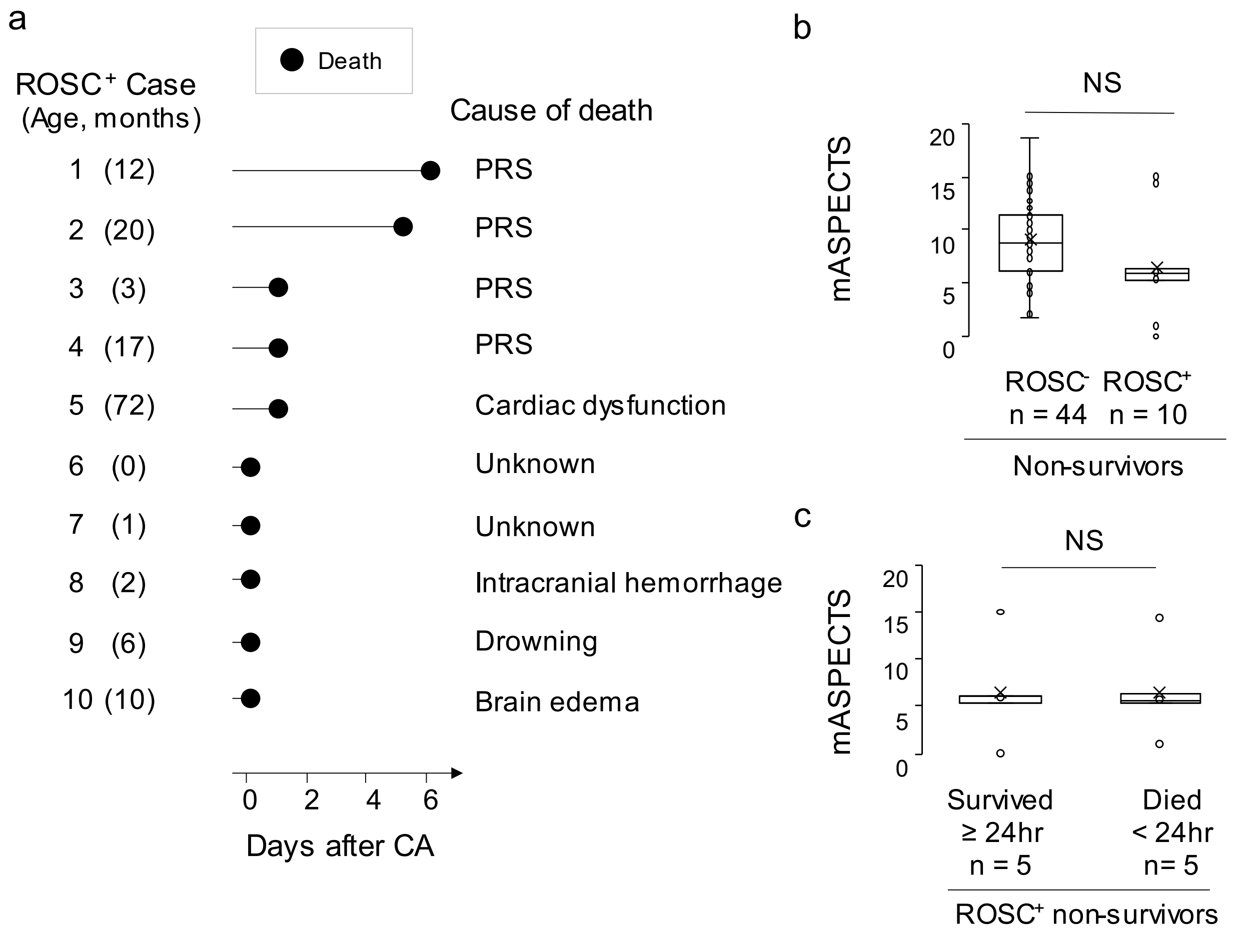

Figure S4. The clinical courses and mASPECTS of 10 non-survivors with ROSC.

(a) Non-survivors with return of spontaneous circulation (ROSC). The Case IDs (1-10) and their ages are shown on the left. The durations of their survival after CA (solid lines) and the day of death (black circles) are shown in the middle. Causes of death are noted on the right. Note that 5 of these patients survived for ≥24 h but died by 6 days after CA. PRS, post-resuscitation syndrome.

(b) The mASPECTS of the 54 non-survivors. No significant difference in the mASPECTS was observed between those with ($ROSC^+$) and without ROSC ($ROSC^-$). p=0.067, Mann-Whitney U-test.

(c) The mASPECTS of the $ROSC^+$ non-survivors. No difference was observed in the mASPECTS between the subgroups of non-survivors who died within 24 h and those who survived ≥24 h after CA. p=1, Mann-Whitney U-test

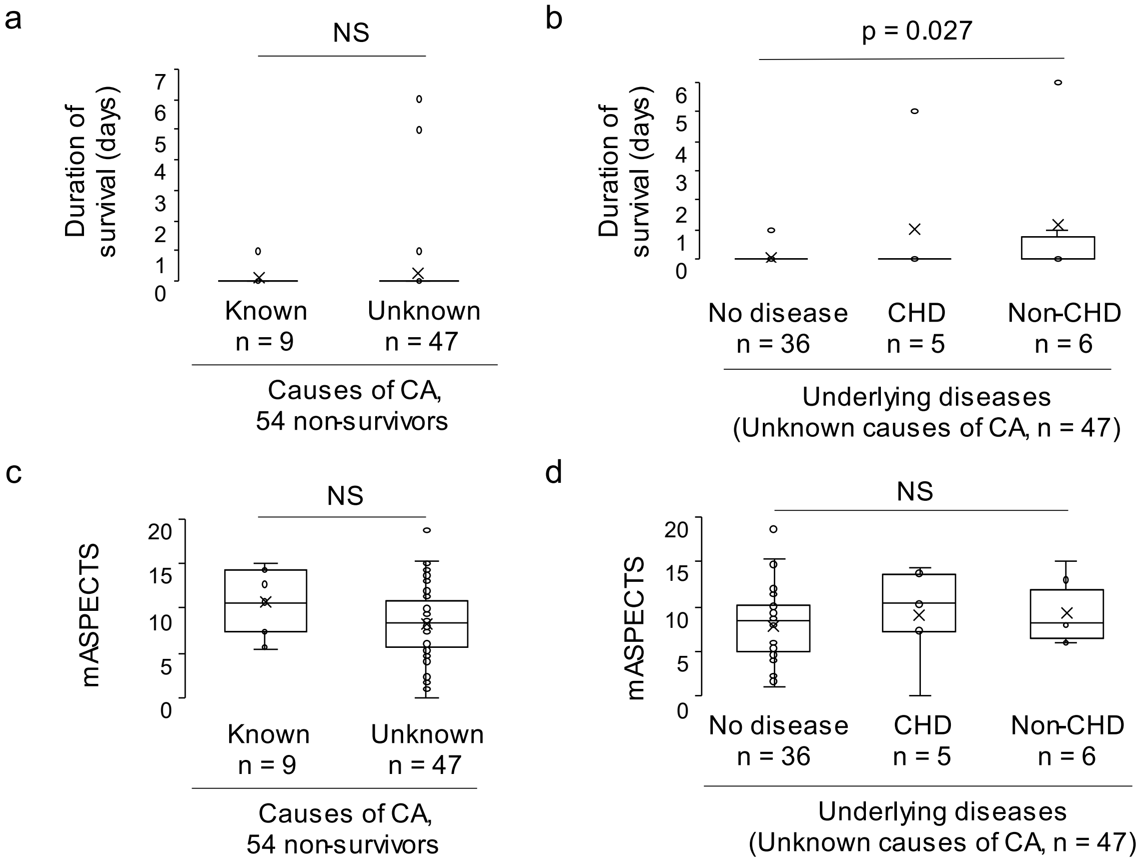

Figure S5. Heterogeneous backgrounds for unknown causes of CA.

(a) No marked differences were noted in the survival duration between the subgroups of non-survivors (n=54) with and without known causes of CA (n=9 and 47, respectively). p=0.857, Mann-Whitney U-test. NS, not significant. For panels (c) and (d), the same criteria were applied for the subcategories of non-survivors with and without known causes of CA as shown in panels (a) and (b).

(b) The survival duration of non-survivors without known causes of CA (n=47). These patients were subclassified according to their underlying diseases: CHD (n=5), non-CHD (n=6) and no diseases (n=36). The box-dot plots show a longer survival among patients with underlying diseases of non-CHD than among those without underlying diseases. p=0.0272, Kruskal-Wallis test.

(c) The comparison of mASPECTS between the non-survivors with and without known causes of CA. No marked difference was observed in this parameter. p=0.110, Mann-Whitney U-test.

(d) The comparison of the mASPECTS among the patients with the indicated subcategories of underlying diseases. p=0.663, Kruskal-Wallis test

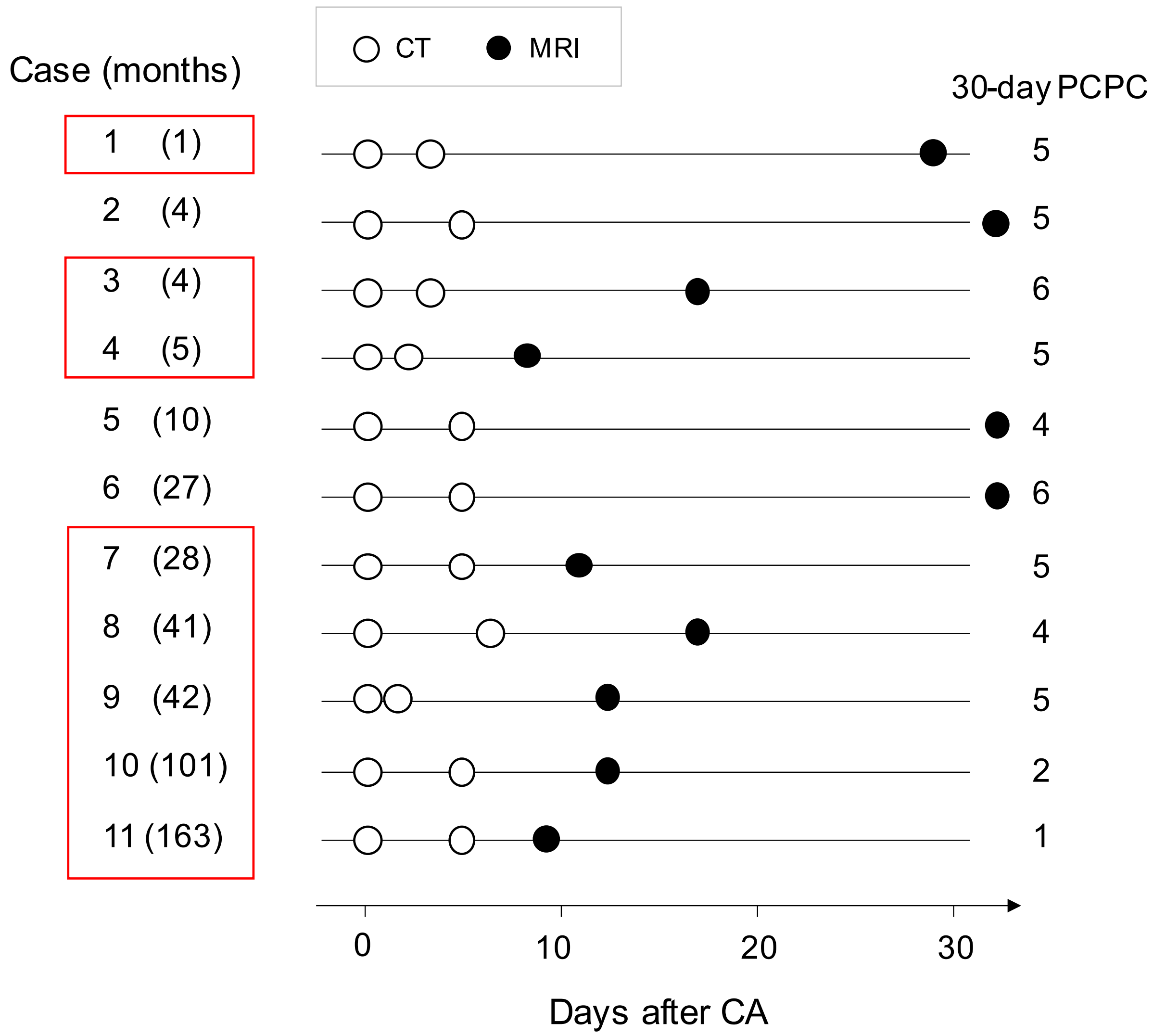

Figure S6. The 30-day survivors with MRI data.

Among 14 survivors, 11 underwent the second CT (white circles) and follow-up MRI (black circles). Eight of them (shown in red squares) received MRI prior to 30 days after CA. Case IDs (#7 and #11) are identical to those presented in Figure 5a. PCPC scores on day 30 are noted on the right end.

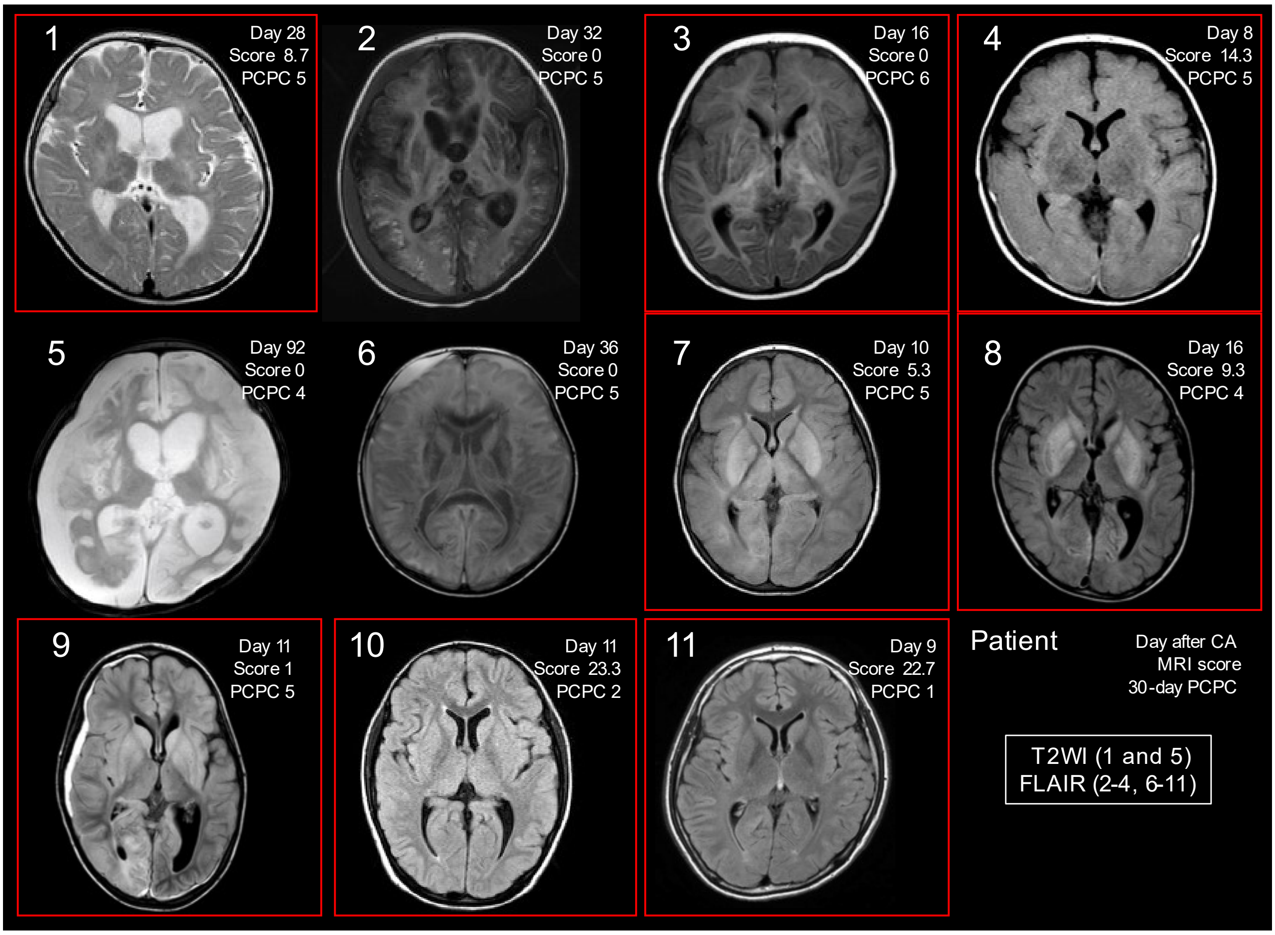

Figure S7. Available MRI data for eleven survivors.

Panels show FLAIR or T2-weighted images of 12 survivors. Case IDs are shown on the left top of each panel. On the right top, days after CA, MRI score (quantitatively analyzed brain damages on T2, FLAIR and DWI), and 30-day PCPC are shown. Red squares highlight 8 patients who received MRI within 30 days after CA.

Table S1. Causes of cardiac arrest

	Survived >30 days n=14	Died ≤30 days n=56	p-value
Shockable rhythm[#]	3 (21.4)	1 (1.8)	0.023
Drowning	2 (14.3)	3 (5.3)	0.260
Intracranial hemorrhage	2 (14.3)	1 (1.8)	0.100
Sudden infant death syndrome	0 (0)	2 (3.6)	1.000
Leukemic emboli	0 (0)	1 (1.8)	1.000
Asphyxia	0 (0)	1 (1.8)	1.000
Unknown	7 (50.0)	47 (83.9)	0.012

The values in parentheses indicate the percentages.

Shockable rhythm means ventricular tachycardia or fibrillation.

Table S2. Pediatric Cerebral Performance Category

Score	Category	Description
1	Normal	Age-appropriate level of functioning
2	Mild disability	Able to interact at an age-appropriate level. Minor neurological disease that is controlled and does not interfere with daily functioning.
3	Moderate disability	Below age-appropriate functioning. Neurological disease that is not controlled and severely limits activities.
4	Severe disability	Excessively dependent on others for provision of activities of daily living
5	Coma/vegetative state	Coma; unawareness.
6	Death	

Table S3. Underlying diseases

	Survived >30 days n=7	Died ≤30 days n=12
Congenital heart disease	1 (14.3)	6 (50.0)
PDA, trisomy 21, hypothyroidism	1	0
ASD, LBWI	0	1
ASD, multiple anomaly	0	1
PS after balloon valvuloplasty, LBWI	0	1
AVSD with CRT, asplenia	0	1
Single ventricle with HOT, LBWI	0	1
TGA, LBWI	0	1
Non-congenital heart disease	6 (85.7)	6 (50.0)
Low birth weight	1	2
Hypertrophic cardiomyopathy	1	0
Cleft lip	1	0
Bradycardia	1	0
Cardiac tumor	1	0
Epilepsy	1	0
Trisomy 21, hypothyroidism, TAM	0	1
History of Kawasaki disease	0	1
Apnea, gastroesophageal reflux	0	1
Hydronephrosis	0	1

The values in parentheses indicate the percentages.
ASD: atrial septal defect, PS: pulmonary stenosis, AVSD: atrioventricular septal defect, CRT: cardiac resynchronization therapy, HOT: home oxygen therapy, TGA: transposition of the great arteries, LBWI: low birth weight infant, PDA: patent ductus arteriosus, TAM: transient abnormal myelopoiesis

Table S4. Causes of death

	Underlying disease		No underlying disease
	CHD	Non-CHD	
	n=6	n=6	n=44
Unknown	5 (83.3)	6 (100)	35 (81.8)
Arrhythmia	1 (16.7)	0 (0)	0 (0)
Drowning	0 (0)	0 (0)	3 (6.8)
Sudden infant death syndrome	0 (0)	0 (0)	2 (4.5)
Intracranial hemorrhage	0 (0)	0 (0)	1 (2.3)
Leukemic emboli	0 (0)	0 (0)	1 (2.3)
Asphyxia	0 (0)	0 (0)	1 (2.3)
Pneumonia	0 (0)	0 (0)	1 (2.3)

The values in parentheses mean the percentages.

CHD: congenital heart disease

Table S5. Factors for survival over 30 days after cardiac arrest assessed by a multivariable analysis

Variables	Total, n	Death, n (%)	Crude OR	p-value	Adjusted OR*	p-value
≥12 months or older, yes	20	13 (65.0)	3.31 (0.98-11.2)	0.054	2.15 (0.38-12.10)	0.387
Underlying disease, yes	19	12 (63.2)	3.67 (1.08-12.5)	0.038	0.86 (0.13-5.58)	0.874
<1-h interval from the last obs. to CPR, yes	24	14 (58.3)	7.14 (1.93-26.5)	0.003	4.64 (0.79-27.20)	0.089
Witness of collapse, yes	11	7 (63.6)	2.80 (0.69-11.4)	0.151	0.42 (0.06-3.22)	0.407
Bystander CPR, yes	42	33 (78.6)	1.25 (0.37-4.23)	0.715	1.07 (0.23-4.95)	0.928
Shockable rhythm detected by EMS, yes	3	0 (0)	>100 (0.00-∞)	0.993	>100 (0.00-∞)	0.993
Known causes of cardiac arrest, yes	16	9 (56.3)	5.22 (1.47-18.5)	0.011	1.57 (0.27-9.04)	0.611

ORs (95% confidence intervals) and p-values were obtained from a logistic regression analysis.
Adjusted values were obtained from a logistic regression analysis after adjusting for all variables listed in this table.
CPR: cardiopulmonary resuscitation, OR: odds ratio

Table S6. **Clinical outcomes of the patients with underlying diseases**

Pt	age/sex Months	Underlying disease	PCPC pre-onset	PCPC post-30 days	Causes of CA	Causes of Death	ECG rhythm at the time of detection/admission	sGWR	mASPECTS
1	101/M	PDA, trisomy 21	2	2	unknown	no	non-SR/ROSC	1.15	22.3
2	0/M	Cleft palate	1	5	unknown	no	non-SR/ROSC	1.02	6.3
3	10/F	Cardiac tumor	1	4	SR	no	SR/ROSC	1.19	14.3
4	28/M	Epilepsy	1	5	drowning	no	non-SR/ROSC	1.10	8.0
5	41/M	LBWI	1	4	drowning	no	non-SR/ROSC	1.10	13.0
6	63/M	Bradycardia	1	1	SR	no	SR/ROSC	1.21	22.7
7	163/M	HCM	1	1	SR	no	SR/ROSC	1.30	21.7
8	1/F	ASD, LBWI	1	6	unknown	unknown	non-SR/non-SR	1.05	7.33
9	8/M	TGA, LBWI	1	6	unknown	unknown	non-SR/non-SR	1.12	10.3
10	12/F	PS, LBWI	1	6	unknown	unknown	non-SR/non-SR	1.10	13.7
11	14/M	ASD, multiple anomaly	3	6	unknown	unknown	non-SR/non-SR	1.09	14.3
12	20/M	Single ventricle, LBWI, HOT	3	6	unknown	unknown	non-SR/non-SR	1.03	0
13	72/M	AVSD, asplenia	1	6	SR	SR	non-SR/SR	1.16	15.0
14	2/M	LBWI	1	6	unknown	unknown	non-SR/non-SR	1.00	15.0
15	4/M	Trisomy 21, TAM	3	6	unknown	unknown	non-SR/non-SR	1.11	8.3
16	4/M	Hydronephrosis	1	6	unknown	unknown	non-SR/non-SR	1.16	8.0
17	12/M	Apnea, GER	1	6	unknown	unknown	non-SR/non-SR	1.17	6.0
18	15/M	Kawasaki disease	1	6	unknown	unknown	non-SR/non-SR	1.07	13.0
19	17/M	LBWI	1	6	unknown	unknown	non-SR/non-SR	1.08	6.0

Shockable rhythm means ventricular tachycardia or fibrillation. PCPC: Pediatric Cerebral Performance Category, CA: cardiac arrest, M: male, F: female, PDA: patent ductus arteriosus, SR: shockable rhythm, ROSC: return of spontaneous circulation, LBWI: low-birth-weight infant, HCM: hypertrophic cardiomyopathy, ASD: atrial septal defect, TGA: transposition of the great arteries, PS: pulmonary stenosis, HOT: home oxygen therapy, AVSD: Atrioventricular septal defect, TAM: transient abnormal myelopoiesis, GER: gastroesophageal reflux
